# Obstetric Intensive Care Admissions and Neonatal Outcomes: 15 Years of Experience from a Single Center

**DOI:** 10.3390/medicina60121937

**Published:** 2024-11-25

**Authors:** Filipa Ramalho Rocha, Tiago Neto Gonçalves, Maria Inês Xavier-Ferreira, Francisco Laranjeira, Gonçalo Meleiro Magalhães, Maria Inês Lopes, Marta Sousa, Daniela Pestana, Élia Fernandes, Ana Chung, Ana Berdeja, Gonçalo Cassiano Santos, Natália Marto, António Messias, Jorge Lima

**Affiliations:** 1 Internal Medicine Department, Hospital da Luz Lisboa, 1500-650 Lisboa, Portugal; filipa.rocha@hospitaldaluz.pt (F.R.R.); tiago.neto.goncalves@hospitaldaluz.pt (T.N.G.); francisco.soares.laranjeira@hospitaldaluz.pt (F.L.); nfmarto@hospitaldaluz.pt (N.M.); 2 High-Risk Pregnancy Center, Hospital da Luz Lisboa, 1500-650 Lisboa, Portugal; esfernandes@hospitaldaluz.pt (É.F.); achung@hospitaldaluz.pt (A.C.); aberdeja-ext@hospitaldaluz.pt (A.B.); goncalo.cassiano.santos@hospitaldaluz.pt (G.C.S.); 3 Faculdade de Medicina, Universidade de Lisboa, 1649-028 Lisboa, Portugal; 4 Intensive Care Department, Hospital da Luz Lisboa, 1500-650 Lisboa, Portugal; maria.xavier.ferreira@hospitaldaluz.pt (M.I.X.-F.); goncalo_meleiro@hotmail.com (G.M.M.); maria.ines.lopes@hospitaldaluz.pt (M.I.L.); marta.sousa@hospitaldaluz.pt (M.S.); amessias@hospitaldaluz.pt (A.M.); 5 Neonatology Department, Hospital da Luz Lisboa, 1500-650 Lisboa, Portugal; maria.jardim.pestana@hospitaldaluz.pt; 6 Obstetrics and Gynecology Department, Hospital da Luz Lisboa, 1500-650 Lisboa, Portugal; 7 Comprehensive Health Research Center, NOVA Medical School, Faculdade de Ciências Médicas, NMS, FCM, Universidade NOVA de Lisboa, 1169-056 Lisboa, Portugal

**Keywords:** critical illness, high-risk pregnancy, maternal morbidity, maternal mortality

## Abstract

*Background and Objectives:* Maternal severe morbidity and mortality are measures for assessing maternal healthcare, and admissions to the intensive care unit (ICU) can be used to study these metrics. Here, we analyze ICU admissions of pregnant or postpartum women in a tertiary hospital. *Materials and Methods:* This is a retrospective, single-center, observational cohort study of obstetric intensive care admissions at a Portuguese hospital spanning 15 years. We analyzed maternal, admission-related, and neonatal variables from women admitted during pregnancy and up to 42 days post-delivery. *Results:* We identified 150 obstetric ICU admissions (0.9% of all ICU admissions, with an admission rate of 4.4 per 1000 deliveries). The mean age was 34 years, with most women being multiparous and 16.7% utilizing assisted reproductive technology. Notably, 45% of the women were above 35 years old. Most (86.7%) were admitted during the early postpartum period after undergoing a cesarean section (74%). The most frequent reasons for ICU admission were postpartum hemorrhage and hypertensive disorders of pregnancy. The mortality rate was 1.3%. The mean gestational age of newborns was 36 weeks and 6 days, and 46.4% were admitted to the neonatal ICU. We recorded one fetal death at 25 weeks and no neonatal deaths. *Conclusions:* The unique needs of obstetric ICU patients emphasize the need for specialized training of multidisciplinary teams. Severe postpartum hemorrhage was responsible for significant morbidity and disability, prompting a reassessment of delivery practices.

## 1. Introduction

Maternal mortality is defined as ‘the death of a woman while pregnant or within 42 days of termination of pregnancy, irrespective of the duration and site of the pregnancy, from any cause related to or aggravated by the pregnancy or its management’ [1]. It has long been regarded as an indicator of the quality of maternal healthcare by health authorities and, consequently, is the subject of ongoing monitoring.

Despite global improvements in healthcare and declines in maternal mortality rates at the beginning of this century, certain developed countries have noted a reversal of this trend, with maternal mortality rates now increasing [2,3]. The most recent data from the United Kingdom show a mortality ratio of 10.9 per 100,000 live births, with an increased risk of death among Black and Asian women [2]. In Portugal, the latest data are consistent with this trend, with maternal mortality ratios reaching 12.8 and 20.1 per 100,000 live births in 2018 and 2020, respectively [4,5]. While this may partially be due to improvements in notification systems, a thorough examination of this trend is indeed necessary. The Portuguese National Health Authority report on maternal mortality identified Black race, multiple pregnancies, maternal age above 35 years, and pregnancies in women with pre-existing comorbidities as factors contributing to the increase in morbidity [4]. These risk factors are becoming increasingly prevalent in developed countries due to migration trends and shifts in the profile of pregnant women. It has been recognized that maternal age above 35 years, and especially above 40 years, is an independent risk factor for both maternal and neonatal adverse outcomes [6].

Severe acute maternal morbidity, or near misses, refers to severe events in pregnant or postpartum women that nearly lead to death, providing a valuable tool for studying obstetric healthcare [7,8]. Despite some limitations, the admissions of obstetric patients to intensive care units (ICU) can serve as a proxy for studying these events, acting as an indicator of clinical severity and an indirect measure of maternal healthcare quality [8,9,10]. On an institutional level, studying ICU obstetric admissions can offer revealing insights into the needs of the local population and the performance of the institution. These data can be used to adjust hospital protocols, which will lead to improved maternal and fetal outcomes [7,10].

The objective of this study is to deliver an all-inclusive characterization of pregnant and postpartum women admitted to the ICU in a Portuguese tertiary hospital over 15 years.

## 2. Material and Methods

This study was conducted at Hospital da Luz Lisboa, a large tertiary private hospital that manages over 3000 deliveries annually. The adult ICU comprises 32 beds, with 16 level II beds and 16 level III beds. Level II care is indicated for patients who require close monitoring or intervention, while level III care is reserved for those needing advanced respiratory support or support for at least two organ systems. Admission is based on availability and is open to all women requiring critical care or close monitoring.

We reviewed the electronic health records of consecutive women who required admission to the intensive or intermediate care unit during pregnancy or up to 42 days post-delivery from 2007 to 2022 (15 years). No exclusion criteria were used.

Data were extracted from hospital and ICU databases, then collected and pseudonymized into a Microsoft Excel file for data analysis. The search involved retrieving all women under 55 years from the ICU database. Obstetric-related ICU admissions were subsequently identified from the coded admission diagnosis and pregnancy or postpartum status.

The descriptors included were separated into maternal and neonatal characteristics. Maternal data encompassed demographic information, medical history, obstetric history, and variables linked to the ICU admission episode, such as admission reason, origin, duration of stay, and destination. Neonatal data included gestational age at birth, birth weight, Apgar score, ICU admission, need for respiratory support, and location on the seventh day.

Statistical analysis was performed using Microsoft Excel and the Statistical Package for Social Sciences (SPSS) v26.0 software. Parametric data are presented as means and standard deviations. Categorical variables are described using absolute and relative frequencies [n (%)]. Normality was tested with the Kolmogorov–Smirnov test. The Chi-square test or Fisher’s exact test was used to analyze categorical variables, as appropriate, and the Mann–Whitney test was used for continuous variables. A *p*-value of less than 0.05 was considered statistically significant.

## 3. Results

Over the 15-year study period, the ICU received 150 obstetric admissions involving 148 patients out of 34,360 deliveries. One patient was readmitted twice during the same pregnancy, first for an intrabdominal abscess following an appendectomy and later for a uterine rupture. Obstetric ICU admissions during the study period accounted for approximately 0.9% of total adult admissions, with an admission rate of 4.4 per 1000 deliveries.

The mean age of the admitted women was 34 years, ranging from 21 to 54 years. Notably, 45% of these women were aged over 35. Older patients showed higher rates of admission due to postpartum hemorrhage (PPH) (*p* = 0.004) and had more previous pregnancies (*p* = 0.034) and cesarean sections (*p* = 0.07) compared to patients under 35 years. However, there was no significant difference in hypertensive diseases between the two groups. Complete demographic data are presented in Table 1.

Thirty-four patients (40%) were overweight, with the maximum recorded body mass index (BMI) being 41.8 kg/m^2^. Fifty-four women (36.5%) had at least one previous medical condition, primarily thyroid dysfunction, thrombophilia, diabetes, hypertension, and asthma. Twenty-four patients (16.2%) were on chronic medication, typically hormone supplementation, antithrombotic, or antidiabetic drugs. Only five patients (3.4%) were active smokers.

In terms of obstetric history, 46 patients (31.1%) were nulliparous. Thirty-eight women (25.7%) had experienced at least one miscarriage, and 32 (21.6%) had undergone a previous cesarean section (CS).

Regarding the current pregnancy (Table 2), 20 cases involved assisted reproductive technology (ART), and there were 19 multiple pregnancies. Thirty-eight women (25.7%) experienced complications during the current pregnancy, particularly gestational hypertension, gestational diabetes, and placental abruption.

There were 135 deliveries at our hospital, representing 95.7% of our cohort, with 7 deliveries occurring after ICU admission. More than half of the women (58.8%) delivered without labor, and 74% underwent a cesarean section, with half of those being urgent. Six women experienced pregnancy loss.

The motive for hospital admission was delivery for 58 patients (38.6%). The remainder were admitted for pregnancy-related complications, most commonly hypertensive disorders (Table 3).

Table 4 summarizes the characteristics of ICU admissions. Most women (86.7%) were admitted in the postpartum period, particularly within the first 48 h following delivery. Among these, more than half were at term (gestational age at delivery > 37 weeks), and 10 patients delivered beyond 40 weeks of gestation.

Twenty hospital admissions (13.3%) occurred during pregnancy, primarily in the second trimester (45%), with a mean gestational age of 16 weeks. Thirty-five percent of the women were admitted during the first trimester, and 20% during the third trimester.

The majority of patients were transferred to the ICU from the delivery ward (34%), the operating room (26%), or the emergency department (ED), specifically obstetric ED (22%) and general ED (12.6%).

Most admissions were due to obstetric causes (74.7%). The most common diagnoses upon ICU admission were postpartum hemorrhage (PPH) (n = 58; 38.7%) and hypertensive disorders of pregnancy (n = 52; 34.7%), followed by infectious, cardiovascular, and neoplastic disorders.

The most frequent cause of PPH was uterine atony, occurring in 16 patients. Among these patients, 86.2% had anemia, and 58.6% had coagulopathy. Nineteen patients (32.8%) underwent emergency peripartum hysterectomy, 93% required transfusion of blood-derived products, and 24% needed vasopressor support.

Regarding hypertensive disorders of pregnancy, there were 28 cases of preeclampsia with severe features and 17 cases of HELLP syndrome. Further, two instances of posterior reversible encephalopathy syndrome (PRES) were identified, one presenting as eclampsia. Of these women, 90.4% received a magnesium sulfate infusion, and 25% required intravenous antihypertensive medication.

Infectious diseases were varied and included urinary tract infection, malaria, bacterial meningitis, influenza, typhoid fever, intra-abdominal infection, and suppurative thrombophlebitis. Cardiovascular disorders comprised cases of supraventricular arrhythmia, carotid artery dissection, and peripartum cardiomyopathy. Neoplastic disorders predominantly involved patients admitted in the immediate post-operative period after tumor resection. These included three cases of central nervous system tumors, one case of renal angiomyolipoma, and one case of tonsillar neoplasm.

Interventions during the ICU stay included transfusion of blood-derived products in 44% of patients, administration of magnesium sulfate in 36%, intravenous antihypertensive medication in 9.3%, vasopressor support in 5.3%, and mechanical ventilation in 4.7% of cases. One patient required plasmapheresis for acute pancreatitis caused by severe hypertriglyceridemia.

The mean length of stay in the ICU was 2.2 days, with most patients (88.7%) transferred to the obstetric ward. The observed mortality rate was 1.3%. In our unit, there was one death due to postpartum bleeding caused by placenta previa. Additionally, we included in our mortality analysis a 37-week pregnant woman with severe hypertriglyceridemic acute pancreatitis who was transferred to another ICU, where she subsequently died.

A total of 140 babies were born to mothers who required intensive care during pregnancy or postpartum, as detailed in Table 5. The mean gestational age was 36 weeks and 2 days, with a prematurity rate of 41.4%. The mean birth weight was 2766 g ± 648 g. Sixty-five newborns (46.4%) were admitted to the Neonatal Intensive Care Unit (NICU), and 14 (10%) required respiratory support. By the seventh day, most of the newborns had been discharged home. No neonatal deaths occurred. However, there was one fetal death at 25 weeks, where the mother was admitted due to hemorrhagic shock from placental abruption.

## 4. Discussion

Our study profiles a cohort of 148 pregnant and postpartum women who were admitted to the ICU at a single center in Portugal over a 15-year duration. Notably, the distribution of admissions throughout the study duration was uneven due to a progressive increase in the number of deliveries at our hospital, which escalated from 331 deliveries in 2007 to 3321 in 2022. In comparison to other cohorts and national registries from developed countries, our patients were typically older and had a lower prevalence of chronic and pregnancy-related diseases. However, there was a higher utilization of ART. Remarkably, despite demographic variations and anticipated social differences in a cohort from private healthcare, the incidence and pattern of ICU admissions were akin to other cohorts, predominantly characterized by early postpartum admissions due to PPH and hypertensive diseases of pregnancy.

This study is one of the few national studies focused on obstetric intensive care admissions and marks the first conducted within a private hospital setting, adding a unique perspective on maternal morbidity in Portugal. We analyzed admissions from the hospital’s inception, capturing several changes in its size, organization, and record system. The number of deliveries at this hospital has steadily risen in recent years, culminating in 3321 deliveries and 3383 live births in 2022. This places it among the foremost maternity hospitals in Portugal [11]. Our study documented 150 admissions during pregnancy and postpartum, representing, to the best of our knowledge, the largest Portuguese cohort reported to date [12,13,14].

In our study, obstetric critical care admissions accounted for 0.9% of total adult ICU admissions. This aligns with the described low prevalence of pregnant and postpartum women requiring admission to ICUs in both developed and developing countries [9,12,13,14,15,16,17,18,19].

We report an incidence rate of 4.4 ICU admissions per 1000 deliveries. A 2010 systematic review by Pollock et al. revealed an overall incidence rate of 2.7 obstetric critical care admissions per 1000 births, ranging from 0.7 to 13.5. This review incorporated studies from both low and high-income countries across primary, secondary, and tertiary centers [9]. National-level studies from France and Australia/New Zealand reported similar incidences of ICU admissions, with rates of 3.7 and 4.8 patients per 1000 deliveries, respectively [17,19]. However, single-center studies performed in tertiary care hospitals in developed countries reported higher ICU admission rates, with 7.4 per 1000 births in the United Kingdom, 9.9 per 1000 births in Austria, and 11.8 per 1000 births in Australia—likely reflecting these hospital’s focus on high-risk obstetric care [16,20,21].

Interestingly, three recent studies of Portuguese cohorts reported obstetric critical care incidence rates of 0.7, 1.59, and 2.8 per 1000 births, which were lower than our own [12,13,14]. ICU admission for obstetric patients is influenced by various factors, including the structure of the healthcare system, the availability and accessibility of ICU and high-dependency unit beds, and institutional practices such as utilizing delivery wards for surveillance purposes. Therefore, using this parameter as an indicator of severe maternal morbidity may be misleading when comparing countries and healthcare facilities.

Risks for adverse maternal, fetal, and neonatal outcomes progressively increase with age, particularly over 40 years [6]. Older women have a higher risk of maternal complications such as gestational diabetes mellitus, preeclampsia, labor dystocia, and cesarean delivery. They are also at risk of delivering a preterm neonate, requiring NICU admission, and having a low birth weight [6]. The mean age in our cohort was 34 years, with 45% of women older than 35 years. This exceeds the age demographics reported in both national and international studies [9,12,13,14,17,19,21,22]. However, our cohort displayed comparable or even lower rates of chronic diseases, maternal complications, and neonatal complications compared to previous reports [9,12,13,14,16,17,19,21,22].

The prevalence of chronic disease was 36.5%, and obesity was observed in 15.3%; however, significant data on booking BMI was unavailable. As noted in previous studies, the prevalence of chronic disease appears low among obstetric ICU patients, consistent with expectations for a young female population, despite these conditions being recognized as risk factors for adverse outcomes [13,14]. Additionally, our reported rates of gestational hypertension and diabetes were lower than those found in other studies [13,21,22].

The age discrepancy we observed may have contributed to the higher use of ART in our cohort (16.7%), consistent with the ART rate reported for the Austrian cohort described by Foessleitner (12.4%). This cohort also had a higher median age (32 years) [21].

Our cohort consisted primarily of Caucasian women, followed by those of the Black race. Black race has been highlighted as a significant risk factor for maternal morbidity and mortality. Thus, clinicians should heighten their awareness of its importance in healthcare considerations [2,4]. However, ethnicity data were missing for approximately one-third of patients, reflecting current concerns related to racial categorization. This trend holds the potential to affect the interpretation of health data and the tailoring of care for specific population groups.

In regard to obstetric history, we noted a higher proportion of multiparous women (68.9%) than previously reported (32 to 49%) [12,13,14,16,21,22]. This is somewhat unexpected given that primiparity is widely recognized as a risk factor for hypertensive disorders of pregnancy, one of the primary reasons for ICU admission [23].

While we did not analyze socioeconomic factors, we can hypothesize that our cohort of self- or insurance-financed women excludes disadvantaged populations typically included in most published data. However, the variables pertaining to ICU admission mirrored those in other reports. Consistent with the published data, the primary reasons for ICU admission were PPH (38.7%), mainly due to uterine atony, and hypertensive disorders of pregnancy (34.7%) [10,12,13,14,17,19,22,24]. This accounts for the increased frequency of admissions during the peripartum period, particularly within the first 24 h post-delivery. The majority of patients came from the delivery ward or the operating room—a pattern consistent with previously described cohorts [10,14,17,21,24]. Our reported CS rate was notably high among women requiring ICU admission (74.5%) [10,14,16,17,18,21,22]. This CS rate includes women admitted for elective CS who required critical care due to complications arising from the procedure (37%), as well as those who underwent urgent CS due to critical illness (37%).

It is worth noting that a significant portion of women requiring critical care were initially admitted to the hospital for delivery purposes (38.6%), including active labor, labor induction, or elective CS. This group typically comprised previously healthy women experiencing critical illness due to peripartum complications, notably PPH. Our study reveals that the majority of these patients required blood transfusions, with approximately a quarter also needing vasopressor support. Moreover, one-third of these patients underwent a postpartum emergency hysterectomy, aligning with reported rates in the literature [14,16,18,21]. These findings underscore the severity of hemorrhage in our cohort.

Major obstetric bleeding is a leading cause of maternal mortality, especially in developing countries, accounting for 27.1% of maternal deaths worldwide [25]. It remains a consistently predominant reason for ICU admission across cohorts from both developed and developing countries, with its incidence on the rise [19,25].

As reviewed by the International Postpartum Hemorrhage Collaborative Group, several risk factors for PPH have been identified, including increasing age, obesity, CS, labor induction, and multiple pregnancies [26]. The working group proposes that additional factors, such as a more relaxed approach to labor duration, rising obesity rates, or alterations in third-stage labor management, may contribute to the increase in PPH rates [26]. We found higher rates of PHH in women older than 35 years old and in those with more previous pregnancies and prior CS. We can hypothesize that these factors, along with frequent delivery by CS, may contribute to the elevated prevalence of PPH. Given that PPH is a preventable cause of maternal morbidity and mortality, proactive measures are imperative. Training should be provided to all staff involved in maternity care, focusing on blood loss assessment, postpartum monitoring, and the implementation of standardized management protocols.

Pregnancy-related hypertensive disorders were the second most common cause of obstetric ICU admission and the main pregnancy complication leading to hospitalization in our study. Interestingly, only 36.5% of these women were nulliparous, a known risk factor for this condition [23]. A subgroup analysis showed comparable rates of chronic diseases, use of ART, multiple pregnancies, mean age, and BMI when compared with other women in our cohort, suggesting these are universal risk factors for maternal morbidity. Out of the 52 patients in this group, only 14 had a previous diagnosis of gestational hypertension, three had gestational diabetes, and three patients were recorded as taking acetylsalicylic acid (AAS) during pregnancy. The minimal use of AAS, coupled with our reported mean gestational age of 36 + 6 and a mean birth weight of over 2700 g, suggests that the majority of hypertensive disorders were likely late-onset preeclampsia. Almost all patients received a 24 h magnesium sulfate infusion as per hospital protocol, and there was only one case of a convulsive episode in a patient with documented PRES.

Regarding ICU interventions, the rates of mechanical ventilation and vasopressor use were relatively lower than those described in other reports [12,14,18,19,21]. This discrepancy may, in part, be explained by our low threshold for admission and the availability of ICU Level II and Level III beds. Additionally, we did not include in our analysis patients who were admitted to the ICU directly from surgery while still under mechanical ventilation since we did not classify this as an ICU intervention.

Most patients had a brief stay in the ICU, the mean duration being two days. This duration is shorter than what other national studies have reported, but it aligns with findings from international sources [9,12,14,19,21].

Only two women died in the ICU during the study period: one from postpartum bleeding due to placenta previa and one from acute pancreatitis as a result of severe hypertriglyceridemia. These incidents represent a mortality rate of 1.3%. The overall rate of maternal mortality in ICUs varies widely from 0 to 40%, with significantly higher rates in lower-income countries [9]. Our mortality rate is similar to those reported for ICU cohorts from developed countries, which range from 0 to 4.2% [12,14,16,18,20,21,22]. Prompt recognition of illness severity, multidisciplinary team assessment, judicious decision-making concerning ICU admission, and accessibility of care are key factors in maintaining low maternal morbidity and mortality [27].

Infant outcomes in our study were positive, with lower rates of low birth weight, prematurity, NICU admissions, and the need for respiratory support compared to similar studies conducted in developed countries [14,21,22]. Our perinatal mortality rate was also lower than previously reported [9,22,24]. It is worth noting that we adhere to national laws prohibiting the delivery of preterm infants before 32 weeks at private institutions. Consequently, our prematurity rate was relatively low at 41.4%, with most preterm newborns being classified as late preterm. This contributed to an overall favorable prognosis for the infants.

Our study has significant strengths. First, we analyzed a large number of cases, representing, to our knowledge, the largest cohort of this population in our country and among the largest in Europe. Second, our observational period extended over 15 years, which is considerably longer than many studies reported in the existing literature. This extended timeframe enabled us to identify consistency over time regarding ICU admission reasons and outcome measures.

One of the main limitations of our study stems from its retrospective design, which influences our ability to identify all relevant cases and restricts the available information. We attempted to minimize this issue by reviewing all patient files from the records during the study period, but it still resulted in underreporting. Data concerning ethnic background and BMI were frequently missing, which limits our study’s ability to evaluate their effect on maternal morbidity, as obesity and Black race are pointed as risk factors for maternal complications. Secondly, as a private hospital, our population does not accurately represent the entire Portuguese population. Instead, it constitutes a selection of women who are likely more literate and usually have insurance coverage, which may act as protective factors against maternal mortality, as suggested by a WHO multi-country survey on maternal and newborn health and the latest MBRRACE-UK report on maternal deaths and morbidity [2,27]. This aspect may affect the generalizability of our results.

## 5. Conclusions

Our study illuminates the risk factors, causes, and outcomes of severe maternal morbidity resulting in ICU admissions in a large Portuguese maternity hospital and substantially contributes to the existing literature on the subject. We present the largest national cohort to date spanning a 15-year period, with a meaningful proportion of patients above 35 years old and use of assisted reproductive technology.

The limited number of diagnoses associated with ICU admissions emphasizes the need for specialized training for obstetricians, anesthesiologists, and nurses to detect early warning signs of severe PPH and hypertensive disorders of pregnancy. Likewise, in cases where general ICUs admit pregnant and postpartum women, intensive care personnel must have thorough training in the management protocols for these specific conditions.

Over a third of our cohort were women admitted for delivery who developed critical illnesses due to peripartum complications, primarily severe PPH. This resulted in significant morbidity, including nineteen hysterectomies and one death, mirroring the increasing trends of PPH worldwide. We propose reassessing current delivery practices to mitigate PPH risk factors, especially elective labor induction and cesarean sections.

## Figures and Tables

**Table 1:** Demographic and clinical characteristics (n = 148).

Age	
Mean ± SD, years	34 ± 5
Distribution, n (%) <30 years 30–34 years 35–39 years 40–44 years ≥45 years	21 (14.2) 61 (41.2) 46 (31.1) 16 (10.8) 4 (2.7)
Ethnicity (n = 87)	
Distribution, n (%) Caucasian Black Asian Other Missing data	73 (83.9) 10 (11.5) 3 (3.4) 1 (1.2) 61
BMI (n = 85)	
Mean ± SD, kg/m^2^	25 ± 5.3
Obese (≥30 kg/m^2^), n (%) Yes No Missing data	13 (15.3) 72 (84.7) 63
Chronic disease	
Distribution, n (%) Thyroid dysfunction Thrombophilia Diabetes Hypertension Asthma	9 (6.1) 5 (3.4) 5 (3.4) 4 (2.7) 3 (2)
Obstetric history	
Distribution, n (%) Nulliparous Primiparous Multiparous	46 (31.1) 54 (36.5) 48 (32.4)
Prior miscarriage	38 (27.7)
Prior CS	32 (21.6)

SD—standard deviation; CS—cesarean section.

**Table 2:** Current pregnancy and delivery characteristics (n = 148).

Characteristics	n (%)
Conception (n = 120)	
Spontaneous Assisted reproduction Missing	100 (83.3) 20 (16.7) 28
Multiplicity	
Single Multiple	129 (87.2) 19 (12.8)
Disease during pregnancy	
Gestational hypertension Gestational diabetes Placental abruption Other	19 (12.8) 8 (5.4) 3 (2) 8 (5.4)
Pregnancy outcome (n = 141)	
Delivery in the absence of labor Delivery in labor Pregnancy loss Missing	83 (58.8) 52 (36.9) 6 (4.3) 7
Delivery route	
Urgent/Emergent CS Elective CS Vaginal eutocic Vaginal vaccum Vaginal forceps	50 (37) 50 (37) 14 (10.4) 13 (9.6) 8 (6)

CS—cesarean section.

**Table 3:** Main hospital admission motives (n = 150).

Reason for Admission	n (%)
Delivery Elective CS Labor induction Labor	58 (38.6) 24 (16) 20 (13.3) 14 (9.3)
Hypertensive disorders	49 (32.7)
Infection/Sepsis	10 (6.7)
Ruptured ectopic pregnancy	5 (3.3)
Postpartum hemorrhage	5 (3.3)
Third trimester bleeding	4 (2.7)

CS—cesarian section.

**Table 4:** ICU admission characteristics (n = 150).

Timing of Admission	
Postpartum, n (%) Median time after delivery—days Pregnancy, n (%) Median gestational time—weeks	130 (86.7) 1 20 (13.3) 16
Origin, n (%)	
Delivery ward Operating room Obstetric emergency department General emergency department General ward Other	51 (34) 39 (26) 33 (22) 19 (12.6) 4 (2.7) 4 (2.7)
Admission diagnosis, n (%)	
**Obstetric cause** Postpartum hemorrhage Uterine atony Genital tract trauma Retained placenta Placenta previa/accreta Coagulopathy Ruptured ectopic pregnancy Other Hypertensive disorders of pregnancy Preeclampsia with severe features HELLP Preeclampsia without severe features PRES Acute fatty liver of pregnancy	**112 (74.7)** 58 (38.7) 16 (10.7) 11 (7.3) 8 (5.3) 8 (5.3) 7 (4.7) 5 (3.3) 3 (2.1) 52 (34.7) 28 (18.7) 17 (11.3) 5 (3.3) 2 (1.3) 2 (1.3)
**Non-obstetric cause** Infectious disorders Cardiovascular disorders Neoplastic disorders Allergic reaction Surgical disorders Cardiorespiratory arrest Neurological disorders Gastrointestinal disorders Other	**38 (25.3)** 10 (6.7) 7 (4.7) 6 (4) 3 (2) 3 (2) 2 (1.3) 2 (1.3) 1 (0.7) 4 (2.7)
Interventions, n (%)	
Transfusion of blood-derived products Magnesium sulfate IV antihypertensive drugs Vasopressor Mechanical ventilation Plasmapheresis	66 (44) 54 (36) 14 (9.3) 8 (5.3) 7 (4.7) 1 (0.7)
Length of stay	
Mean ± SD—days	2.2 ± 1.8
ICU score, Mean ± SD	
APACHE SAPS-II	8.9 ± 6.4 16.6 ± 12.2
Destination, n (%)	
Ward Home Other hospital	133 (88.7) 10 (6.7) 6 (4)

APACHE—Acute Physiologic Assessment and Chronic Health Evaluation; HELLP—hemolysis, elevated liver enzymes, and low platelets; IV—Intravenous; PRES—posterior reversible encephalopathy syndrome; SAPS—Simplified Acute Physiology Score.

**Table 5:** Newborn characteristics.

Gender, n (%)	
Male Female	84 (60) 56 (40)
Gestational age ^1^	
Mean ± SD—weeks	36 ± 2
Distribution, n (%) Term Late preterm birth Early preterm birth Very early preterm birth Extreme preterm birth	82 (58.6) 50 (35.7) 6 (4.3) 1 (0.7) 1 (0.7)
Birthweight ^2^	
Mean ± SD, grams	2766 ± 648
Distribution, n (%) Appropriate for gestational age Small for gestational age Large for gestational age	86 (61.4) 26 (18.6) 28 (20)
5 min Apgar score, n (%)	
≥7 points ≤3 points	138 (98.6) 2 (1.4)
NICU and interventions, n (%)	
Admission to NICU Respiratory support	65 (46.4) 10 (7.1)
Location at day 7, n (%)	
Home NICU Other hospital	124 (88.6) 15 (10.7) 1 (0.7)

^1^ Term (≥37 wk), late preterm birth (34–36 w), early preterm birth (32–33 w), very early preterm birth (28–31 w), extreme preterm birth (<28 w). ^2^ Appropriate for gestational age (birth weight between the 10th and 90th percentile for gestational age), small for gestational age (birth weight of less than 10th percentile for gestational age), large for gestational age (birth weight above the 90th percentile for gestational age). NICU—Neonatal intensive care unit; SD—Standard deviation.

## Data Availability

The data presented in this study are available on request from the corresponding author. The data are not publicly available due to ethical and privacy restrictions.
